# Neural responses to heartbeats distinguish self from other during imagination

**DOI:** 10.1016/j.neuroimage.2019.02.012

**Published:** 2019-05-01

**Authors:** Mariana Babo-Rebelo, Anne Buot, Catherine Tallon-Baudry

**Affiliations:** a Laboratoire de Neurosciences Cognitives et Computationnelles, Inserm, Ecole Normale Supérieure, PSL University, Paris, France; b Sorbonne Université, Inserm U 1127, CNRS UMR 7225, Institut du Cerveau et de la Moelle Epinière, ICM, Ecole Normale Supérieure, ENS, Centre MEG-EEG, F-75013, Paris, France

**Keywords:** Imagination, Self, Perspective taking, Heartbeat-evoked responses, Interoception, Magnetoencephalography

## Abstract

Imagination is an internally-generated process, where one can make oneself or other people appear as protagonists of a scene. How does the brain tag the protagonist of an imagined scene as being oneself or someone else? Crucially, during imagination, neither external stimuli nor motor feedback are available to disentangle imagining oneself from imagining someone else. Here, we test the hypothesis that an internal mechanism based on the neural monitoring of heartbeats could distinguish between self and other. 23 participants imagined themselves (from a first-person perspective) or a friend (from a third-person perspective) in various scenarios, while their brain activity was recorded with magnetoencephalography and their cardiac activity was simultaneously monitored. We measured heartbeat-evoked responses, i.e. transients of neural activity occurring in response to each heartbeat, during imagination. The amplitude of heartbeat-evoked responses differed between imagining oneself and imagining a friend, in the precuneus and posterior cingulate regions bilaterally. Effect size was modulated by the daydreaming frequency scores of participants but not by their interoceptive abilities. These results could not be accounted for by other characteristics of imagination (e.g., the ability to adopt the perspective, valence or arousal), nor by cardiac parameters (e.g., heart rate) or arousal levels (e.g. arousal ratings, pupil diameter). Heartbeat-evoked responses thus appear as a neural marker distinguishing self from other during imagination.

## Highlights

• Heartbeat-evoked responses differentiate self from other during imagination.
• These effects were located in the precuneus and posterior cingulate.
• The neural monitoring of the heart could be a mechanism for self/other distinction.

## 1 Introduction

Being able to vividly project oneself or other people in imagination is a fundamental process, as it allows us to simulate - and thus be prepared for - future events (Christoff et al., 2016; Schacter et al., 2007). The mechanisms underlying imagination are intriguing (Schacter et al., 2007; Suddendorf et al., 2009), because imagining implies the ability to create mental scenes in the absence of sensory inputs. In particular, how does the brain tag the protagonist of an imagined scene as being oneself or someone else? In externally-driven processes, sensory signals or motor feedback can characterize the self when we hear our own voice (Heinks-Maldonado et al., 2005) or perform a voluntary movement (Blakemore et al., 1999), for example. But critically, since imagination is developed *internally*, none of such signals can help disentangle self from other. Moreover, there is considerable anatomical overlap between the brain regions activated when imagining oneself and those recruited when imagining someone else (Decety and Grèzes, 2006). An additional tagging neural mechanism should thus be implemented to account for the biological bases of the self-other distinction. This mechanism could involve the neural monitoring of internal bodily signals, such as heartbeats, which has recently been proposed to be a marker of the self (Park and Tallon-Baudry, 2014; Tallon-Baudry et al., 2018).

The neural monitoring of heartbeats can be measured experimentally via heartbeat-evoked responses (HERs), i.e. transient changes in brain activity locked to heartbeats. HERs were shown to encode the degree of self-relatedness of spontaneous thoughts (Babo-Rebelo et al., 2016a, 2016b) and were associated with different aspects and levels of bodily self-consciousness (Park et al., 2016; Sel et al., 2016). These results suggest a link between the neural monitoring of the heart and selfhood, such that the brain would refer to internal bodily signals, in particular the heart, to tag mental processes as being self-related. However, previous experiments explored *degrees* of selfhood or used paradigms involving external sensory stimuli. If HERs contribute to a general mechanism for the implementation of the self, they should also distinguish between self and other, during internally-generated imagination. Interestingly, some of the regions that distinguish self from other in perspective taking (Vogeley and Fink, 2003) and in particular during imagination (Ruby and Decety, 2001), i.e. the precuneus and anterior insula, also respond to heartbeats (Babo-Rebelo et al., 2016a, 2016b; Park et al., 2016). Here, we test the hypothesis that heartbeat-evoked responses distinguish between self and other during imagination.

**Fig. 1:**
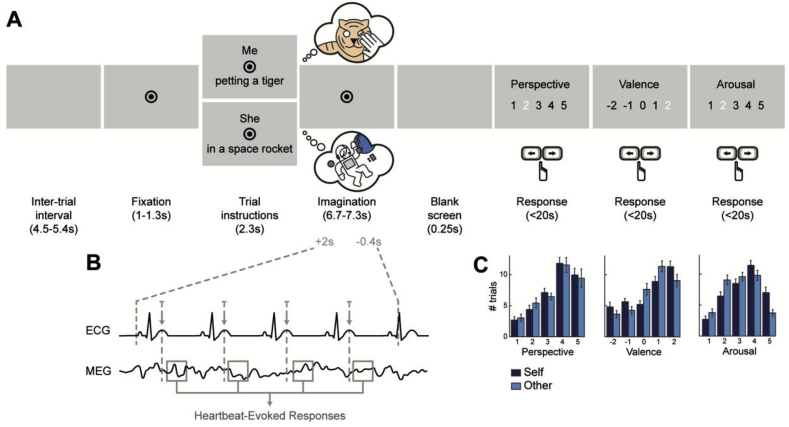
Experimental paradigm and behavior. ***A***, Time course of a trial. At each trial, participants had to imagine the person (Self, i.e. oneself from the first-person perspective, or Other, i.e. a friend from the third-person perspective) in the scenario indicated, until fixation disappeared. They then had to rate the imagined scenario in terms of Perspective (how well they succeeded in adopting the indicated perspective), Valence (how pleasant the scene was) and Arousal (how arousing the scene was). ***B***, Computation of Heartbeat-Evoked Responses (HERs) during the imagination period. T-peaks occurring from 2s after the beginning of the imagination period to 0.4s before the end of this period were selected. MEG data was extracted locked to these T-peaks to compute HERs. ***C***, Distribution of responses for the Perspective, Valence and Arousal scales, for both Self and Other trials, across all participants. Self trials were significantly more arousing than Other trials (paired *t*-test on the average Arousal ratings for Self and Other: p = 0.0005, uncorrected). Error bars indicate SEM.

To test this hypothesis, we recorded both brain activity using magnetoencephalography (MEG) and cardiac activity (electrocardiogram, ECG) while participants imagined themselves or a friend, in a series of cued scenarios (Fig. 1A). Participants imagined themselves from the first-person perspective, from inside their body, and imagined their friend from the third-person perspective, by picturing the friend in the scenario. The scenarios either explicitly cued an action (ex: “petting a tiger”), or indicated a general context (ex: “in a space rocket”). All scenarios were unlikely or unreal, to avoid between-condition differences in memory retrieval and familiarity. After imagining each scenario, participants were asked to rate in 5-point scales how well they were able to adopt the indicated perspective (Perspective scale), the valence of the imagined scenario (Valence scale) and their level of arousal during imagination (Arousal scale). We tested whether the amplitude of HERs during imagination of oneself differed from the amplitude of HERs taking place during imagination of the friend (Fig. 1B).

## 2 Materials & methods

### 2.1 Participants

25 right-handed volunteers were recruited on an online database open to the general community (https://expesciences.risc.cnrs.fr/). Volunteers were French native speakers, students or young professionals and participated in this study after giving written informed consent. They were paid for their participation (80 Euros). The study was approved by the local ethics committee. Participants were screened to exclude cases of prosopagnosia or any cardiac problems. Two participants were excluded from analysis, one because of a noisy electrocardiogram recording, and the other because of an extremely fast heart-rate (mean interbeat interval of 555 ms, >2 SDs faster than the average interbeat interval in the other participants). Twenty-three participants were thus included in the analysis (9 male; mean age: 24.3 ± 0.6).

### 2.2 Experimental procedure

The day before the experiment, participants were asked to choose the friend they would imagine in the task. The friend had to be the same gender and around the same age as the participant. The participant had to know him/her quite well and had to be able to clearly visualize him/her. It could not be someone the participant was romantically involved with, their best friend nor a relative. To assess the closeness of the selected friend, participants filled in a modified version of the Relationship Closeness Inventory (Berscheid et al., 1989) (RCI, excluding questions related to romantic relationships), where total scores range from 3 to 30. The average RCI score among participants was 12.4 ± 0.8, which is intermediate between close (scores around 16) and distant relationships (scores around 9).

Before the MEG recording, participants were given written and oral instructions about the task, as described in the next section. They performed a short practice block (2 trials of each condition), followed by four blocks of 9 trials of each condition (randomly presented), during which MEG and physiological data were acquired. Then, participants performed the heartbeat-counting task, in order to assess their interoceptive abilities (Schandry, 1981). They had to count their heartbeats while fixating the screen, during one practice block followed by five test blocks of different durations (practice block: 45s; test blocks: 60, 80, 100, 120, 140s – order randomized between participants), without feedback on performance. The heartbeat perception score was computed as the mean score over the 5 test blocks. In the same session, participants also performed a trait-judgment task and a resting state recording. These data are not presented here. After the recording session, participants completed a short feedback questionnaire and the Daydreaming Frequency Scale (Giambra, 1993; Stawarczyk et al., 2012).

### 2.3 Imagination task

Each trial (Fig. 1A) began with a fixation mark (central black dot, radius 0.21° of visual angle, surrounded by a black circle, radius 0.52° of visual angle), presented for 1–1.3s on a grey background, followed by the instruction screen. The instruction screen specified the person to imagine (condition Self: “Me”, or condition Other: “He”/”She”), above fixation, and provided a brief description of the scenario to be imagined, below fixation. The instructions remained on screen for 2.3s, after which they disappeared, leaving only the fixation mark. Participants had then to imagine the scenario while fixating, until the fixation mark disappeared, after 6.7–7.3s. The imagination period was variable to limit temporal anticipation processes.

Participants were instructed to adopt a first-person perspective in trials where they had to imagine themselves, meaning they should imagine the scenario from inside their own body. In trials where they had to imagine their friend, participants should adopt a third-person perspective and visualize the friend without interacting with him/her. Participants were also instructed to focus on the imagination of the person rather than on the visual details of the scenario.

The imagination period stopped with a blank screen (0.25s) and was followed by the presentation of the three scales (the order was randomized between participants, but constant for each participant). Participants had to rate on 5-point scales: the perspective (“How well did you manage to adopt the perspective in this scenario?”, from 1: not very well, to 5: very well); the valence (“How pleasant was the scenario?”, from −2: very unpleasant, to 2: very pleasant); and the arousal (“How arousing was the imagined scenario?”, from 1: not arousing, to 5: very arousing) of the imagined scenario. The order of presentation of the responses (ascending or descending) was randomized at each trial, to minimize motor preparation. Participants responded by pressing left and right buttons (index and middle finger respectively) to select the appropriate response. They validated their response with their right thumb, within 20s per scale. A new trial started after an inter-trial interval (blank screen, 4.5–5.4s). The task was displayed on a semi-translucent screen at 85 cm viewing distance and was programmed using PsychToolbox.

### 2.4 Scenarios

An initial list of 78 scenarios, e.g. brief descriptions of the situation to be imagined, was created. Scenarios described unreal or unlikely situations, in order to limit the familiarity or memory confound (scenarios as “having breakfast” for example were not included, because these would be familiar for oneself but less so when applied to someone else). In a pilot behavioral study, 10 subjects performed the imagination task with these scenarios and indicated afterwards which scenarios were difficult to imagine. 72 scenarios were finally selected, 42 of which contained an action verb (examples: “to drive a Formula 1 car”, “to erect a standing stone”). The remaining scenarios did not contain any verb, and rather indicated a context or environment (examples: “in the Middle Ages”, “in the jungle”). Each scenario was presented only once during the experiment. Scenarios assigned to condition Self for subject 1 were assigned to condition Other for subject 2 and vice-versa, for all pairs of subjects. This way, each scenario was associated with “Self” and with “Other” conditions the same number of times across subjects. The proportion of scenarios with and without a verb was distributed equally between conditions.

### 2.5 Recordings

Continuous magnetoencephalographic (MEG) data was acquired using a whole-head MEG system with 102 magnetometers and 204 planar gradiometers (Elekta Neuromag TRIUX, sampling rate of 1000 Hz, online low-pass filtered at 330 Hz). Electrocardiogram data (ECG, sampling rate 1000 Hz, 0.03–330 Hz) was obtained from 7 electrodes placed around the base of the neck and referenced to a left abdominal location. The ground electrode was located on the back of the neck. Two ECG electrodes were placed over the left and right clavicles, two over the top of the left and right shoulders, two over the left and right supraspinatus muscle and one over the upper part of the sternum. Electromyographic activity (EMG, two electrodes on the right cheek, 10–330 Hz) from the right zygomaticus major was acquired in order to control for facial muscle activity, a potential source of noise for MEG data. Indeed, because the scenarios were quite unlikely, participants might occasionally smile or laugh. Horizontal and vertical eye position and pupil diameter were monitored using an eye-tracker (EyeLink 1000, SR research) and recorded together with electrophysiological data.

### 2.6 MEG data preprocessing

Continuous MEG data was denoised using temporal signal space separation (TSSS, as implemented in MaxFilter) and filtered between 0.5 and 45 Hz (4th order Butterworth filter). Large movement or muscle artefacts were visually detected and the corresponding data excluded from analysis. Independent Component Analysis (ICA) as implemented in the Fieldtrip toolbox (version: 20161025) (Oostenveld et al., 2011) was used to correct for the cardiac field artefact, on both magnetometers and gradiometers, based on epochs of −0.2 to 0.2s around the R-peaks of interest devoid of movement, muscle, blink or saccade artefacts. Because TSSS induces rank-deficiency, we defined the number of ICA components by first computing a Principal Component Analysis (PCA). We then removed all independent components with mean pairwise phase consistency (Vinck et al., 2010) with the ECG in the 0–25 Hz range larger than two standard deviations of all components. We iterated this procedure until no outlier components were found or a maximum of two excluded components was reached. A similar ICA procedure was then applied to correct for blink artefacts. Each trial was divided in five segments (from the onset of fixation to the end of the imagination period), devoid of large saccades (>4°), movement or muscle artefacts. Component decomposition was performed on those segments, with the number of components defined by PCA. We then computed the correlation between the time course of each component and the vertical EOG signal. Using an iterative procedure, we rejected components whose correlation coefficient exceeded three standard deviations of all components, until no outlier was present or the maximum of three components to reject was reached. For two subjects the automatic selection of components was ineffective and one subject did not have a vertical EOG recording. For these subjects, the selection of components was done by visually identifying the characteristic topographies of blink ICA components and rejecting those components.

### 2.7 Pupil diameter preprocessing

Pupil diameter was obtained with the Eyelink software (EyeLink 1000, SR research). Blink epochs were automatically identified by the acquisition software. We extended those epochs by 80 ms before and after to ensure that the whole blink event was included. We also identified noise in pupil diameter data (e.g. signal variation larger than 1 in arbitrary units, in a 300 ms time window). Portions of pupil diameter data containing blinks or noise were linearly interpolated and low-pass filtered at 10 Hz (fourth-order Butterworth filter). Pupil data were then epoched from 2 to 4 seconds after the onset of the imagination period. Epochs with more than 30% of interpolated data were excluded from further analysis. The remaining epochs were z-scored. Four subjects were excluded from pupil diameter analysis for having a too low number of remaining trials (<1.5 SD).

### 2.8 Heartbeat-evoked responses (HERs)

R- and T-peaks were detected on derivation lead II of the ECG, except in two subjects for whom T-peaks were not clearly visible on lead II and therefore were detected on lead III. ECG recording was band-pass filtered, between 0.5 and 40 Hz (4th order Butterworth filter). We first detected the R-peaks, by correlating the ECG with a template QRS complex defined on a subject-by-subject basis and by identifying the local maximum within the episodes of correlation larger than 0.7. T-peaks were then detected by first correlating the ECG with a template of the T-peak; second, identifying the local maxima within episodes of correlations above a certain correlation value (adapted for each subject) that followed an R-peak by at most 0.4s. R- and T-peak detection was visually verified in all subjects. R- and T-peaks corresponding to noisy ECG data (movement artefacts) or followed/preceded by extra-systolic events were excluded from analysis.

The T-peaks occurring during the imagination period (from 2 seconds after the beginning of the period, to −0.4s before the end of the imagination period) were used for HER analysis. Epochs (from 0.1s before to 0.4s after the selected T-peaks) contaminated with movement or muscular (in particular of the zygomaticus, recorded with the EMG) artefacts were not included in the analysis. T-peaks which were followed by an R-peak by less than 0.4s were not considered for HER computation to avoid any overlap between the HER window of analysis and the residual R-peak artefact. Artefact-free HERs corresponding to Self and Other trials were computed by averaging across heartbeats magnetometer data low-pass filtered at 30 Hz (4th order Butterworth filter). Only trials for which a behavioral response was recorded for each of the three scales were included in the analysis. The time window retained for analysis was 80–350 ms after the T-peak, during cardiac relaxation when the cardiac artefact is minimal (Dirlich et al., 1997).

On average, 191.3 (±5.86) heartbeats per participant were included for the condition Self and 189.1 (±5.94) for the condition Other, corresponding to 5.3 heartbeats on average per trial. No significant difference was observed in the number of included heartbeats between conditions (paired *t*-test, t_(22)_ = 1.24, p = 0.23, Bayes Factor = 1.45 – anecdotal evidence for H0).

### 2.9 Statistical analyses

The difference in HERs between Self and Other was tested on magnetometer data, in the artefact-free time window 80–350 ms after the T-peak, using a cluster-based permutation *t*-test (Maris and Oostenveld, 2007). This method does not require any a priori on spatial regions or latencies and intrinsically corrects for multiple comparisons in time and space. Briefly, the procedure entails the following processing steps. A paired *t*-test was performed to compare HERs for Self versus Other. Individual samples whose *t*-value was below a threshold (p < 0.05, two-tailed) were clustered together based on temporal and spatial adjacency (with a minimum of 4 neighboring channels). A candidate cluster was characterized by the sum of the *t*-values across the individual samples. To test whether such a cluster could be obtained by chance, we permuted the labels “Self” and “Other” 10,000 times and selected the maximal positive cluster-level statistic and the minimal negative cluster-level statistic at each randomization. The two-tailed Monte-Carlo *p*-value corresponds to the proportion of elements in the distribution of maximal (or minimal) cluster-level statistics that exceeds (or is inferior to) the originally observed cluster-level test statistics. The amplitude of the cluster corresponds to the average of magnetometer data across the sensors and time window showing a significant difference between conditions.

### 2.10 General linear models

General linear models (GLMs) were computed to better characterize the trial-by-trial HER cluster amplitude. Importantly, GLMs assess the unique contribution of each regressor, since the shared variance between regressors is partialled out. In the two GLMs performed, trial-by-trial HER cluster amplitude, the number of heartbeats per trial and regressors (ratings on the scales Perspective, Valence and Arousal) were z-scored prior to GLM computation. The regressor “condition” was coded with the value 1 for Self trials, and the value −1 for Other trials. An additional regressor corresponding to the trial number was introduced in the GLM on HER cluster amplitude, in order to group HERs belonging to the same trial. GLMs were computed for each subject, and the betas corresponding to each regressor of interest were tested against zero, over subjects.

### 2.11 Bayes factor computation

Bayes factors were systematically computed, in particular to evaluate evidence in favor of the null hypothesis relative to evidence in favor of the alternative hypothesis. In the case of paired tests, we computed the maximum log-likelihood of the model in favor of the “null” hypothesis and the model in favor of the “effect” hypothesis. The group-level random-effect Bayes factor was computed with a Gaussian distribution corresponding to an effect differing from 0 under a *t*-test with a *p* value of 0.05. We then used the Bayesian information criterion to compare the two models and computed the corresponding Bayes factor.

Bayes factors for correlations and unpaired t-tests were computed using an online calculator tool (http://pcl.missouri.edu/bayesfactor, with the version 0.9.8 of the BayesFactor package, R version 3.3.2 (2016-10-31) on i386-redhat-linux-gnu), based on Jeffrey-Zellner-Siow priors.

Here, BF > 1 supports the null hypothesis and conversely BF < 1 is in favor of the alternative. The corresponding BF interpretation was done according to (Kass and Raftery, 1995).

### 2.12 Surrogate heartbeats

To test whether the observed effects were truly locked to heartbeats, we checked whether differences between Self and Other trials could be obtained with a sampling of neural data that was desynchronized from heartbeats (Park et al., 2014). We created 1000 permutations of heartbeats, where the timings of the heartbeats of trial *i* in the original data were randomly assigned to trial *j*. The same analysis was performed, with the same criteria for rejecting artefactual epochs and computing HERs. For each permutation, we obtained a set of neural responses to surrogate heartbeats and computed the cluster summed t-statistics as described above. For each permutation we extracted the smallest negative sum of t-values, and compared the distribution of those surrogate values with the observed original (negative) sum of t-values.

### 2.13 Anatomical MR acquisition and preprocessing

An anatomical T1 scan was acquired for 22 out of 23 participants. Segmentation of the data was processed with automated algorithms provided in the FreeSurfer software package (Fischl et al., 2004) (http://surfer.nmr.mgh.harvard.edu/). Segmentations were visually inspected and edited when necessary. The white-matter boundary was determined using FreeSurfer and was used for subsequent minimum-norm estimation.

### 2.14 Source reconstruction

We reconstructed sources of HERs occurring from 2 to 4s after the onset of the imagination period. Source reconstruction and surface visualization were performed with the BrainStorm toolbox (version 13-Dec-2016, on Matlab R2012b) (Tadel et al., 2011). For the participant who did not have an anatomical scan, we warped the ICBM152 anatomical template (http://bic.mni.mcgill.ca/ServicesAtlases/ICBM152NLin2009) to fit the shape defined by the digitized head points obtained before MEG acquisition. After co-registration between the individual anatomy and MEG sensors, cortical currents were estimated using a distributed model consisting of 15,002 current dipoles from the combined time series of magnetometer and gradiometer signals using a linear inverse estimator (weighted minimum-norm current estimate, signal-to-noise ratio of 3, whitening PCA, depth weighting of 0.5) in an overlapping-spheres head model. Dipole orientations were constrained to the individual MRIs. Cortical currents were then averaged over the HER time windows for which a significant difference between Self and Other was identified in sensor space, spatially smoothed (FWHM 7 mm) and projected to a standard brain model (ICBM152, 15,002 vertices).

Reliable differences in dipole current values were identified using the cluster-based procedure (first-level p-value: 0.01, 1 neighboring vertex, 1000 randomizations) applied to the 15,002 vertices, as described for the sensor level analysis. The obtained Monte-Carlo *p*-value was thus intrinsically corrected for multiple comparisons across vertices.

Analyses at the source level in a given region of interest were carried by extracting the data of each vertex and flipping the sign of vertices that differed from the main sign of the region. This was achieved using Brainstorm functions for sign flipping. To select the vertices corresponding to the insula, we used the masks from (Deen et al., 2011) corresponding to the ventral anterior, dorsal anterior and posterior insula bilaterally and transformed them to fit the anatomical template of Brainstorm, using the function ImCalc of SPM12. Differences between Self and Other over time, for each insular region of interest, were assessed by computing a cluster-based permutation test, over time (first-level *p*-value: 0.05; number of randomizations: 1000). This method generated candidate clusters, for which we computed Cohen's d effect sizes.

### 2.15 Cardiac measures

Interbeat intervals (IBIs) consisted of the average time distance between T-peaks in the imagination period. The heart rate variability (HRV) corresponded to the standard deviation of the interbeat intervals.

### 2.16 Arousal and emotion intensity controls

Trials in the Self condition were rated as more arousing than trials in the Other condition. To verify that the HER difference was not due to a difference in perceived arousal, we employed a stratification procedure which consists in removing trials until we obtain two groups of trials in the Self and Other condition with similar arousal ratings. The stratification procedure ran as follows. In each subject, the mean arousal rating was computed for Self and Other. One of the two conditions was then randomly selected. If the mean rating of the selected condition was larger than the mean rating of the non selected condition, the trial corresponding to the largest arousal rating of the selected condition (or a trial randomly chosen among all trials having the maximum arousal rating) was excluded. Conversely, if the mean arousal rating of the selected condition was smaller, the trial corresponding to the smallest arousal rating (or a trial randomly chosen among all trials having the minimum rating) was excluded. This procedure was iterated until the difference in arousal ratings between conditions was smaller than 2%. Overall, 61.35 (±1.47) out of 72 trials were included (minimum number of trials included: n = 46; maximal: n = 69), and the final number of trials included did not differ between conditions (paired *t*-test: t_(22)_ = 0.066, p = 0.95, Bayes Factor (BF) = 4.12 – substantial evidence for H0). We then tested whether cluster amplitudes still differed between conditions in this subset of trials for which arousal was equated.

Another measure of emotion intensity was derived from valence ratings, by considering the absolute value of valence. Emotion intensity ratings were stratified using the same procedure as described above, with an equivalent final number of trials included in each condition (mean number of trials included: 64.13 ± 1.48; minimum: n = 42; maximal: n = 71; paired *t*-test on the final number of trials included between conditions: t_(22)_ = -0.24, p = 0.81, BF = 3.96 – substantial evidence for H0).

### 2.17 Data and code availability statement

The data that support these findings and the code used for stimulation and analysis are available from the corresponding author upon request.

## 3 Results

### 3.1 Behavioral results

The distributions of ratings on the Perspective, Valence and Arousal scales for the Self and Other conditions are presented in Fig. 1C. Mean ratings did not differ between Self and Other conditions in the Perspective or Valence scales (Perspective: mean Self: 3.6 ± 0.1 SEM, mean Other: 3.5 ± 0.1, paired *t*-test Self x Other, t_(22)_ = 0.7, p = 0.5, Bayes Factor (BF) = 2.80 – anecdotal evidence for H0; Valence: Self: 3.5 ± 0.1, Other: 3.5 ± 0.1, t_(22)_ = -0.5, p = 0.6, BF = 3.36 – substantial evidence for H0; uncorrected p-values). Imagining oneself was rated as being significantly more arousing than imagining the friend (Arousal: Self: 3.4 ± 0.1, Other: 3 ± 0.1, t_(22)_ = 4.10, p = 0.0005, BF = 0.0018 – decisive evidence for H1; uncorrected p-value). In addition to the arousal effect, Self trials were rated as being more extremely valenced (positively or negatively) than Other trials (absolute value of valence ratings, or emotion intensity; Self: 1.30 ± 0.036, Other: 1.14 ± 0.049, t_(22)_ = 3.65, p = 0.0014, BF = 0.0067 – decisive evidence for H1).

Feedback questionnaires assessed the ease or difficulty in performing the task. About half the participants found that imagining the self was easier than imagining the other (n = 11), while the other half participants (n = 12) had the opposite evaluation. When asked if they were able to imagine the self from the first person perspective (on a 4-level scale), 10 participants reported ‘yes’ and 13 reported ‘rather yes’ (no participants reported ‘no’ or ‘rather no’). When asked if they were able to visualize the friend, 14 participants reported ‘yes’, 8 participants ‘rather yes’ and 1 participant ‘rather no’. Overall, participants were able to easily perform the task.

### 3.2 HER amplitude differs between self and other

We compared the amplitude of HERs occurring during imagination of self with the amplitude of HERs occurring during imagination of other, for heartbeats (T-peaks) occurring between 2s after the onset of the imagination and 0.4s before the end of the imagination period (Fig. 1B). We excluded the beginning of the imagination period to make sure participants already started imagining the scenario.

**Fig. 2:**
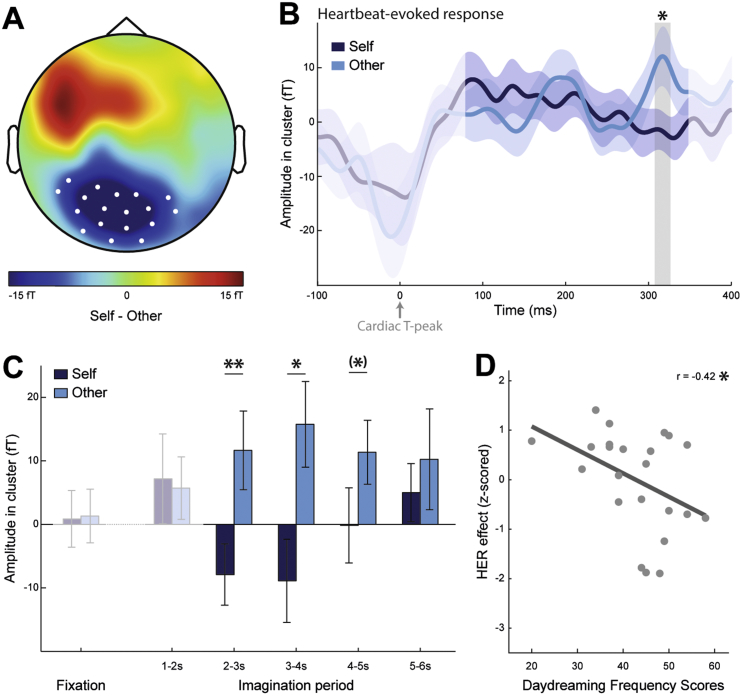
Differential Heartbeat-Evoked Responses (HERs) for imagining oneself (Self) or a friend (Other). ***A***, Topographical map of the HER difference between Self and Other conditions, grand-averaged across 23 participants, in the 307–326 ms time window in which a significant difference was observed (Monte-Carlo p = 0.012). White dots represent the sensors contributing to the significant cluster. ***B***, Time course of the HER (±SEM) for Self and Other, averaged over the sensors marked in white in A. The signal that might be residually contaminated by the cardiac artefact appears in lighter color and was not included in the epoch analyzed. The grey area represents the time window in which a significant difference was observed. ***C***, Temporal evolution of the HER effect, during the imagination period. Amplitude in cluster corresponds to the average brain activity in the T-peak locked time window and sensors revealing a significant HER effect (sensors indicated in A, time window indicated in B). Cluster amplitude was computed for HERs occurring during fixation (1–1.3s), and during the imagination period divided in five windows of 1 second (1–2s, 2–3s, 3–4s, 4–5s, 5–6s). The largest cluster amplitude differences between Self and Other were observed in the windows 2–3s and 3–4s. ***D***, Correlation between the size of the HER effect and Daydreaming Frequency Scores (p = 0.049). HER effect was computed for each participant as the difference between the HER cluster amplitude for Self minus the HER cluster amplitude for Other, z-scored across participants, for HERs in 2–4s of imagination period. Each dot represents one participant. **: p < 0.01, *: p < 0.05, (*): p < 0.1.

Our whole-brain, whole time-window analysis of HERs showed that HERs significantly differed over posterior sensors (Fig. 2A), in the time window 307–326 ms after the T-peak (Fig. 2B; cluster sum(t) = -733.713, Monte-Carlo p = 0.012).

We then looked at the temporal evolution of the effect, during the imagination period, in time windows of 1 second (Fig. 2C). The difference between Self and Other was most pronounced for T-peaks occurring in the time window from 2 to 4 seconds after the onset of imagination. We thus retained this interval for further analysis, although a two-way repeated measures ANOVA on the cluster amplitude, with the factors Condition (Self, Other) and Timing (2–3, 3–4, 4–5, 5–6 seconds) as factors, did not reveal a significant interaction between Condition and Timing (main effect of Condition: F_(1, 22)_ = 11.81, p = 0.0024; main effect of Timing: F_(3, 66)_ = 0.33, p = 0.80; interaction: F_(3, 66)_ = 1.41, p = 0.25).

Because the scenarios could indicate a context or directly cue an action through a verb, we verified that the effect was present in both types of scenarios (paired *t*-test between Self and Other, for context scenarios: t_(22)_ = -2.31, p = 0.031; for action scenarios: t_(22)_ = -3.08, p = 0.0055). In addition, there was no main effect of the type of scenario nor any interaction between the scenario type and the condition (two-way repeated measures ANOVA on the cluster amplitude, with factors Condition (Self, Other) and Scenario type (context, action): main effect of Condition: F_(1, 22)_ = 11.25, p = 0.0029; main effect of Scenario Type: F_(1, 22)_ = 2.62, p = 0.12; interaction: F_(1, 22)_ = 0.40, p = 0.53).

We next tested whether the HER difference between Self and Other was related to the ratings participants provided on a trial-by-trial basis on the success in adopting the perspective, the valence and the arousal of the imagined scenario. To test the unique contribution of the condition (Self/Other), the scales and a possible interaction between them, we performed a general linear model (GLM) for each subject, where HER cluster amplitudes were predicted by 8 regressors: the condition, the ratings on each of the three scales, the interaction between condition and each scale ratings and the trial number. Only the betas corresponding to the regressor “condition” were significantly different from zero across subjects (*t*-test against zero for each β value corresponding to each regressor: Condition: β = −0.21, t_(22)_ = -2.72, p = 0.013, BF = 0.078 – strong evidence for H1; Perspective ratings: β = −0.047, t_(22)_ = -1.76, p = 0.092, BF = 0.60 – anecdotal evidence for H1; Valence ratings: β = 0.017, t_(22)_ = 1.00, p = 0.33, BF = 2.02 – anecdotal evidence for H0; Arousal ratings: β = 0.013, t_(22)_ = 0.70, p = 0.49, BF = 2.88 – anecdotal evidence for H0; Condition*Perspective: β = 0.027, t_(22)_ = 1.59, p = 0.13, BF = 0.82 – anecdotal evidence for H1; Condition*Valence: β = 0.015, t_(22)_ = 0.92, p = 0.37, BF = 2.26 – anecdotal evidence for H0; Condition*Arousal: β = −0.0046, t_(22)_ = -0.27, p = 0.79, BF = 3.92 – substantial evidence for H0). This indicates that HER cluster amplitude variations are uniquely explained by the person who is being imagined.

### 3.3 Control for arousal and emotion intensity effects

Self trials were rated as being more arousing than Other trials. Although the GLM analysis showed that arousal ratings did not modulate the difference in HERs between Self and Other, we ran two additional controls.

First, we considered the possibility that pupil diameter, which is thought as a physiological index of arousal (Murphy et al., 2011), is a more precise marker than the subjective arousal ratings participants provided. Although pupil diameter significantly correlated with arousal ratings, shared variance between the two arousal measures was only 3.2% (*t*-test against zero for Fisher-z-transformed Pearson correlation coefficients between arousal ratings and pupil diameter averaged over 2–4 seconds across subjects: r_(17)_ = 0.18, t_(17)_ = 4.26, p = 0.00047). In the time window 2–4 seconds, where the HER effect was stronger, we did not find conclusive evidence for a difference in pupil diameter between Self and Other (t_(18)_ = 1.65, p = 0.12, BF = 0.75 – anecdotal evidence for H1).

We then further tested arousal as rated by the subjects at each trial. We performed a stratification procedure where some trials were rejected so as to cancel out the difference in arousal ratings between Self and Other (paired *t*-test between average arousal ratings for Self vs Other, before stratification: t_(22)_ = 4.09, p = 0.0005; after stratification: t_(22)_ = 0.14, p = 0.89, BF = 4.09 – substantial evidence for H0). Even after canceling out the difference in arousal ratings, the difference in HER cluster amplitude between Self and Other remained robust (cluster amplitude difference between conditions before stratification: t_(22)_ = -3.25, p = 0.0036; after stratification: t_(22)_ = -2.99, p = 0.0068, BF = 0.04 – strong evidence for H1). Importantly, the size of the HER effect was identical before and after stratification (*t*-test on cluster amplitude before and after stratification: t_(22)_ = -0.43, p = 0.67, BF = 3.60 – substantial evidence for H0).

The stratification procedure equated the arousal ratings between conditions by decreasing the average arousal for Self (paired *t*-test on the mean arousal ratings before vs after stratification, t(22) = 4.06, p < 10^−3^) and increasing the average arousal for Other (t(22) = -3.64, p = 0.0015). Simultaneously, the average rating on the perspective scale decreased for Self (paired *t*-test on the mean perspective ratings before vs after stratification, t(22) = -3.81, p < 10^−3^) and increased for Other (t(22) = 2.87, p = 0.0089), but did not result in a significant difference between conditions (paired *t*-test on the mean perspective ratings for Self vs Other after stratification on arousal, t(22) = -0.76, p = 0.46, BF = 2.72 – anecdotal evidence for H0). This suggests that more arousing trials were also trials where participants adopted the perspectives more successfully. The fact that the HER difference between Self and Other was not affected by the stratification procedure suggests that the HER effect is not linked to the overall success in adopting a perspective nor the associated arousal effect, but rather to the fact that a certain perspective is adopted.

We applied the same stratification procedure on emotion intensity, and found that the HER effect persists when emotion intensity ratings are equalized (paired *t*-test between emotional intensity ratings for Self vs Other, before stratification: t_(22)_ = 3.65, p = 0.0014; after stratification: t_(22)_ = 0.17, p = 0.87, BF = 4.05 – substantial evidence for H0; HER cluster amplitude difference between conditions after stratification: t_(22)_ = -2.90, p = 0.0083, BF = 0.05 – strong evidence for H1). These additional controls confirm that differential arousal levels between Self and Other trials cannot explain the HER effects.

### 3.4 HER effects and inter-subject variability

We tested whether the amplitude of the HER difference between Self and Other correlated with participants' tendency to daydream in their daily lives, as assessed with a questionnaire (Giambra, 1993; Stawarczyk et al., 2012) after the experiment. We found a negative correlation between the daydreaming scores and the HER effect size, measured as the difference between HER cluster amplitude for Self and HER cluster amplitude for Other (Fig. 2D; daydreaming frequency scores: 42.74 ± 1.82; Pearson correlation with the z-scored effect sizes: r_(21)_ = -0.42, r^2^ = 0.172, p = 0.049). This means that people who are used to daydreaming more have a larger HER difference between self- and other-imagination.

This effect was not associated with the ease in performing the task, as assessed with feedback questionnaires. In particular, the amplitude of the HER difference did not differ depending on the ease in imagining the self between subjects (two-sample *t*-test on the HER amplitude difference for the group of participants reporting being ‘able’ to imagine the self versus the group of participants reporting being ‘rather able’ to imagine the self, unpaired *t*-test, t(21) = -0.84, p = 0.41, BF = 2.04 – anecdotal evidence for H0), nor depending on the ease in imagining the friend (two-sample *t*-test on the HER amplitude difference for the group of participants reporting being ‘able’ to imagine the other versus the group of participants reporting being ‘rather able’ to imagine the other, unpaired *t*-test, t(21) = -0.13, p = 0.89, BF = 2.61 – anecdotal evidence for H0).

We also tested whether the size of the HER effect correlated with individual interoceptive abilities, as measured with the heartbeat counting task (Schandry, 1981). We found no evidence that interoceptive abilities modulate the amplitude difference between HERs for Self and HERs for Other (heartbeat perception scores: 0.76 ± 0.029; Pearson correlation with the z-scored effect size: r_(21)_ = 0.038, r^2^ = 0.001, p = 0.86, BF = 2.61 – anecdotal evidence for H0).

We finally tested whether the observed HER effects correlated with general cardiac activity, such as IBI and HRV, as assessed during the following resting state sequence. We found no significant correlation between the amplitude difference in HERs during task and resting state IBI (r_(21)_ = 0.17, r^2^ = 0.029, p = 0.44, BF = 2.10 – anecdotal evidence for H0), nor resting state HRV (r_(21)_ = 0.26, r^2^ = 0.068, p = 0.23, BF = 1.52 – anecdotal evidence for H0).

### 3.5 HERs in the anterior precuneus and posterior cingulate cortex are responsible for these effects

**Fig. 3:**
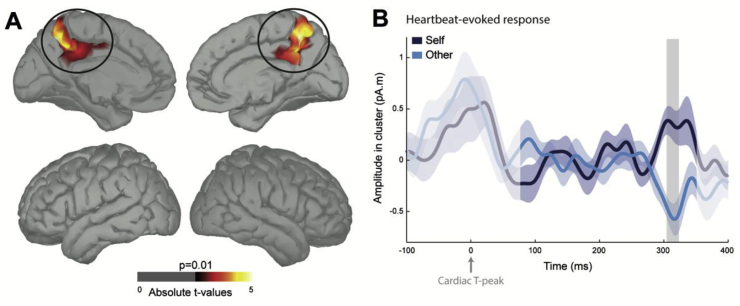
Neural sources of the Heartbeat-Evoked Response (HER) effects. ***A***, HER differences between Self and Other were localized in the anterior precuneus and posterior cingulate cortex bilaterally (left: Monte-Carlo p = 0.022; right: Monte-Carlo p = 0.016; threshold for visualization: >30 contiguous vertices at uncorrected *p* < 0.010). ***B***, Time course of the HERs (±SEM) in the region in A. The signal that might be residually contaminated by the cardiac artefact appears in lighter color. The grey area represents the time window in which a significant difference was observed at the sensor level.

To identify the regions generating the differential HERs, we reconstructed HER sources for Self and Other, averaged the reconstructed neural currents in the time window where we found an effect (307–326 ms after the T-peak) and performed a cluster-based permutation test over all 15,002 vertices to compare activations for Self and Other. The differential HER amplitude was generated in a bilateral region centered on the posteromedial cortex, more precisely in the anterior precuneus and extending ventrally to the posterior cingulate cortex (Fig. 3A and B; Table 1; Left: cluster sum(t) = 482.01, Monte-Carlo p = 0.022, mean Cohen's d = 0.71 ± 0.009, cluster surface 22.82 cm^2^; Right: cluster sum(t) = -600.39, Monte-Carlo p = 0.016, mean Cohen's d = −0.74 ± 0.008, cluster surface 26.27 cm^2^).

**Table 1:** Anatomical description of the source clusters showing significant differential HERs (Fig. 3A).

AAL regions	Peak *t*	Size (cm^2^)	MNI coordinates (peak *t*)		
			X	Y	Z
**Cluster left hemisphere**					
Precuneus	4.99	10.12	−10	−58	50
Mid-cingulate	4.07	10.67	−1	−45	41
Posterior cingulate	2.96	1.20	−1	−44	29
**Cluster right hemisphere**					
Precuneus	−4.77	16.57	9	−50	42
Mid-cingulate	−4.02	7.63	16	−47	35
Posterior cingulate	−3.65	1.35	1	−51	29
Superior parietal gyrus	−2.87	0.066	14	−54	61

### 3.6 Region of interest analysis of the insula

The insular region did not come out as significant from the whole brain analysis. To probe this region further, we conducted a region of interest analysis of the insular cortex by extracting HER source data from three sub-regions of the insula (posterior, dorsal anterior and ventral anterior, bilaterally) as defined in (Deen et al., 2011), and tested for differences in HER amplitude between Self and Other over time, across the whole time window of interest (80–350 ms relative to the T-peak). The clustering procedure identified some candidate clusters with a significant difference between Self and Other in the regions considered, but none of these clusters survived correction for multiple comparisons over time (Supplementary Figure; left: all |cluster sum(t)|<42, all Monte-Carlo p > 0.25; right: all |cluster sum(t)|<62, all Monte-Carlo p > 0.15; uncorrected p-values across multiple sub-regions tested).

We then compared the effect sizes within these candidate clusters with the effect sizes within the posteromedial region showing significant differences in HERs. Mean Cohen's d across vertices in the left precuneus/posterior cingulate cluster was 0.71 ± 0.009 (ranging from 0.59 to 1.04), and for the right homologue was −0.74 ± 0.008 (ranging from −0.59 to −0.99). In the left insula, the largest mean Cohen's d among candidate temporal clusters was 0.51. In the right insula, the largest mean Cohen's d among candidate temporal clusters was 0.57 (absolute values).

Thus, the insula region does not show any reliable difference in HERs. The few candidate temporal clusters show effect sizes that are 23–28% smaller than the effect sizes observed for the precuneus/posterior cingulate region. This indicates that the insula is not generating HERs that reliably distinguish Self and Other.

### 3.7 Test of cardiac parameters during self-vs other-imagination

To account for possible concomitant differences in cardiac parameters, we compared the mean interbeat-interval (IBI) and heart-rate variability (HRV) in the time window 2–4s of imagination between Self and Other conditions. We found no evidence for a difference in IBIs (IBI for Self: 853.93 ms ± 24.81; IBI for Other: 853.41 ± 24.48; t_(22)_ = 0.25, p = 0.81, BF = 3.96 – substantial evidence for H0), nor HRV (HRV for Self: 48.98 ± 2.54; HRV for Other: 53.14 ± 3.75; t_(22)_ = -1.82, p = 0.082, BF = 0.53 – anecdotal evidence for H1).

In addition, we tested whether the features of the imagined scenarios, in terms of success in perspective taking, valence and arousal, could influence heart rate and hence the number of heartbeats observed during the imagination period. There was no significant correlation between number of heartbeats and ratings on any of the scales, for neither Self- nor Other-trials (Pearson correlation between number of heartbeats and scale responses across trials, for each subject, *t*-test against 0 of the Fisher z-transformed coefficients, all p > 0.33, all BF > 2.62 – at least anecdotal evidence for H0). We also computed a GLM where the number of heartbeats was predicted by the regressors condition and the ratings on each of the three scales. None of the regressors was associated with a beta significantly differing from 0 (all p > 0.48, all BF > 3.61 – substantial evidence for H0), which again indicates that the number of heartbeats is not related to the three measured features of the imagined scenarios.

### 3.8 These effects are time-locked to heartbeats and of neural origin

To show that this effect was truly locked to heartbeats and not driven by slow fluctuations of neural activity differing between conditions, we permuted heartbeat timings between trials 1000 times and performed the same analyses on these surrogate heartbeats at the sensor level. Only 4 out of 1000 permutations led to a cluster *t* statistic larger (in absolute values) than the original one, which indicates that our effect is an *evoked-response* to heartbeats, with a Monte-Carlo p value of 0.004.

Heart contractions generate electrical currents that create a magnetic field, which is directly picked up by MEG sensors. In order to show that our results are not due to the electrical activity of the heart but to brain activity, we compared the electrical activity of the heart as measured with the ECG between Self and Other trials, in the time window where we find the HER results. We did this for each of the seven vertical and horizontal ECG leads acquired, to best estimate possible heart-to-MEG signal propagations. We could not find any evidence for a significant difference in heart electrical activity between conditions (paired *t*-test between mean Self vs Other ECG amplitude averaged over 307–326 ms relative to the T-peak in 2–4s of the imagination period: all |t_(22)_|<1.79, all uncorrected p > 0.086; all BFs ≥ 1.15, anecdotal (4 tests) or substantial (10 tests) evidence for H0).

## 4 Discussion

### 4.1 Main results

We hypothesized that the distinction between self and other during imagination could involve an internal mechanism based on the neural monitoring of heartbeats. Our results confirmed this hypothesis, by showing that the amplitude of heartbeat-evoked responses (HERs) in the anterior precuneus and posterior cingulate cortex differed between imagination of self and imagination of a friend. This effect was independent from the success in adopting the perspective, the valence or arousal of the imagined scenario. We also observed that participants who daydream more in their daily lives had larger HER amplitude differences between self and other. We controlled that the HER effects were not due to the cardiac-field artefact nor related to cardiac parameters (IBI, HRV or number of heartbeats included), but rather due to changes in the amplitude of a *neural* response to an internal stimulus, the heartbeat.

Overall, these results are in accordance with previous studies showing a link between HERs and the self (Babo-Rebelo et al., 2016a, 2016b; Park et al., 2017, 2016; Sel et al., 2016) and thereby support the proposal that the neural monitoring of internal bodily signals could constitute a mechanism to tag mental processes as being self-related (Babo-Rebelo et al., 2016a, 2016b; Babo-Rebelo and Tallon-Baudry, 2018; Park et al., 2014; Tallon-Baudry et al., 2018). Our results further show that HERs encode spatial reference frames linked to *who* the active protagonist is, oneself or someone else.

### 4.2 The HER effect is generated in regions associated with the self and with visuo-spatial transformation

The regions most reliably contributing to the HER difference between self and other in this task were the anterior precuneus and posterior cingulate cortex (PCC) bilaterally. HERs related to the self have been observed in the PCC and ventral precuneus (vPrec) region in the context of full-body illusions (Park et al., 2016) and according to the involvement of the self as the agent in spontaneous thoughts (Babo-Rebelo et al., 2016a, 2016b). The posterior medial cortex itself is a large territory comprising several sub-regions (Babo-Rebelo et al., 2016b; Leech and Sharp, 2014) and it would be beyond the resolution of MEG to attempt to refine the spatial analysis. Still, results from three different experiments converge at highlighting the PCC/vPrec as a source of self-related HERs, in agreement with the activation of this region for self-related cognition in fMRI (Qin and Northoff, 2011). The latencies observed here are congruent with the effect reflecting the self in spontaneous thoughts (Babo-Rebelo et al., 2016a), while a much earlier HER effect was observed in the full-body illusion experiment (Park et al., 2016). The reasons for such discrepancies are unknown, but variability can be suspected at all stages, from the transduction of cardiac inputs, that can take place at different latencies in the cardiac cycle (Bishop et al., 2011), to the anatomo-functional routes relaying cardiac information to the PCC/vPrec which remain undetermined.

In this experiment, the differential HER effect is not limited to the PCC/vPrec, but extends to the more dorsal portion of the anterior precuneus. Both animal electrophysiology (Chen et al., 2013; Snyder et al., 1998) and human fMRI literature (Bernier and Grafton, 2010) have underlined the key role of this region in spatial transformations involving different frames of reference, and in particular in the establishment of the egocentric frame of reference during mental imagery (Bicanski and Burgess, 2018). This interpretation in terms of an egocentric frame of reference is reminiscent of our proposal that subjective experience requires a unified egocentric frame of reference based on neural responses to visceral inputs (Babo-Rebelo and Tallon-Baudry, 2018; Tallon-Baudry et al., 2018).

### 4.3 HERs signal who the protagonist is: oneself or someone else

Transient HERs were observed for both self and other, in the same regions but with opposite polarities, indicating that they originate from different neural populations. This is reminiscent of recent findings showing that the same region can encode the value of the reward delivered to the self or delivered to someone else, but in different neurons (Noritake et al., 2018). It is also consistent with the fMRI literature showing a strong overlap between self- and other-processing regions (Legrand and Ruby, 2009), since the BOLD signal is insensitive to the polarity of neural responses. The fact that HERs were also present in the third-person perspective condition associated with the friend indicates that this mechanism is not restricted to fluctuations within the self, as in previous experiments (Babo-Rebelo et al., 2016a; Park et al., 2016). Here, HERs signal *who* the active protagonist is, oneself or someone else, in the imagined scenario. In more mechanistic terms, HERs could index the type of spatial frame of reference adopted for imagination, body-centered for self or world-centered for other.

### 4.4 Other factors associated with self and other conditions

The Self and Other conditions differed along two factors. The first factor was the spatial perspective adopted for imagination. The second factor was that imagining oneself could potentially increase interoceptive attention, e.g. attention to bodily signals including heartbeats.

In this task, self and other conditions were systematically associated with distinct spatial perspectives, first-person perspective for self, third-person perspective for other. Participants reported that both conditions were easy to imagine, suggesting that the different spatial perspectives did not imply different complexity levels. We chose not to ask participants to imagine the friend from the first-person perspective because that would be unnatural, nor the self from the third-person perspective because that would target a more distantiated self (Kross et al., 2005) thereby potentially reducing the contrast between self and other (Christian et al., 2015). Importantly, self and other are intrinsically associated with first- and third-person perspectives respectively. The first-person perspective can only be truly experienced for self, and the friend can only be truly imagined from the third-person perspective. Such association between self/other and perspective is also present in other domains of perspective taking, such as theory of mind (Vogeley et al., 2001), or empathy (Zaki and Ochsner, 2012), which have already been related to interoception (Ernst et al., 2013; Ondobaka et al., 2017). Future studies might investigate potential HER effects in these other domains of perspective taking. This could be particularly relevant in the case of autism, which has been associated with impairments in visuo-spatial perspective taking (Hamilton et al., 2009), theory of mind (Frith, 1997) and empathy (Charman et al., 1997) but also in interoceptive processing (Quattrocki and Friston, 2014).

Imagining oneself might entail increased attention to bodily signals including heartbeats. If attention to interoceptive signals was involved, one would expect the results to be larger in participants with good interoceptive abilities, which was not the case. Note that we employed the heartbeat counting task, that has been recently criticized (Desmedt et al., 2018; Ring and Brener, 2018; Zamariola et al., 2018) – unfortunately after we collected the data. Enhanced interoceptive attention would also modulate HER amplitude in the insula (Canales-Johnson et al., 2015; Pollatos et al., 2005), a region that has been hypothesized to integrate interoceptive inputs with cognitive processing thereby generating a sense of self (Craig, 2009). In the present experiment, the region of interest analysis of the insular cortex did not reveal any differences in HER amplitude between self- and other-imagination, ruling out the enhanced interoceptive attention hypothesis.

## 5 Conclusion

To conclude, our results suggest that HERs could be a mechanism to disentangle self from other, during an entirely internal mental process such as imagination. Heartbeats would work as an internal signal integrated by the brain to tag a mental process as being related to the first-person self or related to someone else. The involvement of the posterior cingulate cortex and ventral part of the precuneus is in agreement with previous studies linking the self and HERs, and the additional involvement of the dorsal part of the anterior precuneus underlines the importance of visuo-spatial transformations and of spatial frames of reference in the distinction between imagining oneself and imagining someone else.

## Contributions

M.B-R. and C.T-B. designed the study. M.B-R. acquired the data. M.B-R., A.B. and C.T-B. analyzed the data. M.B-R. and C.T-B. wrote the paper.

## Funding sources

M.B.-R. was supported by a grant from the Fundação para a Ciência e a Tecnologia SFRH/BD/85127/2012 and by the French program “Investissements d’avenir” ANR-10-IAIHU-06. This work was supported by funding from the European Research Council under the European Union's Horizon 2020 research and innovation program (grant agreement No 670325, Advanced grant BRAVIUS) and a senior fellowship from the Canadian Institute for Advanced Research program in Brain, Mind & Consciousness to C.T.-B., as well as from ANR-17-EURE-0017. The neuroimaging facility (CENIR) where the recordings took place has received funding from the French programs “Investissements d’avenir” run by the “Agence Nationale de la Recherche” (grant number ANR-10-IAIHU-06 and ANR-11-INBS-0006).
