# Supplementary material for: Neural responses to heartbeats distinguish self from other during imagination
Source: Neuroimage. 2019 May 1;191:10–20. doi: 10.1016/j.neuroimage.2019.02.012 (PMC6503945; doi:10.1016/j.neuroimage.2019.02.012)
Supplement: Multimedia component 1 [file mmc1.doc]

**Supplementary Material**


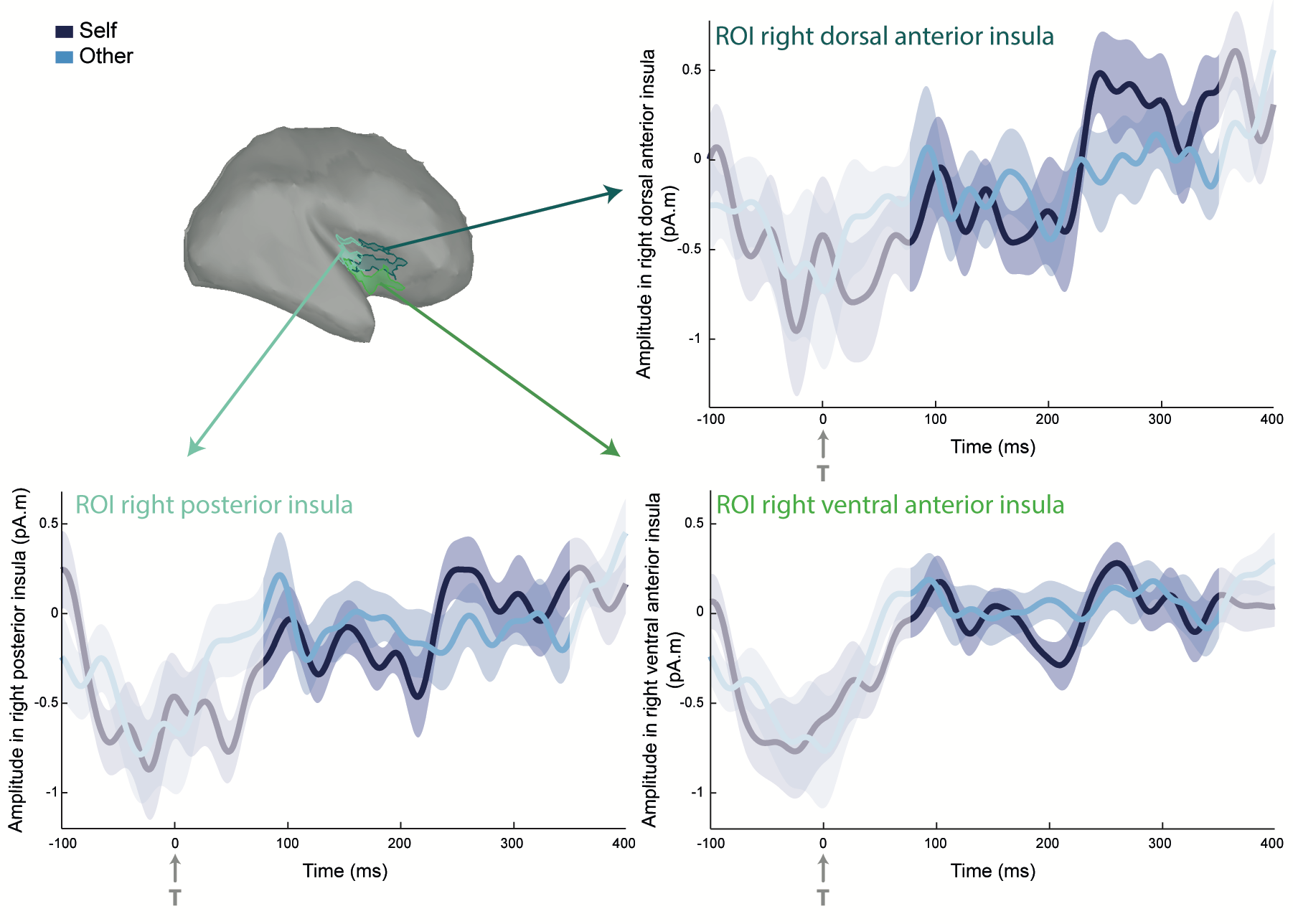


**Supplementary figure**: Heartbeat-Evoked Response (HER) in the three sub-regions of the right insular cortex. No significant differences were observed between HERs during self- vs other-imagination.
